# Environmental Influences on Young Adult Drinking

**Published:** 2004

**Authors:** Alexander C. Wagenaar, Traci L. Toomey, Kathleen M. Lenk

**Affiliations:** Alexander C. Wagenaar, Ph.D., is professor of epidemiology and health policy research at the University of Florida College of Medicine, Gainesville, Florida. Traci L. Toomey, Ph.D., is an associate professor, and Kathleen M. Lenk, M.P.H, is a coordinator, both at the School of Public Health, Division of Epidemiology and Community Health, at the University of Minnesota, Minneapolis, Minnesota

**Keywords:** young adult, underage drinking, undergraduate student, alcoholic beverage, AOD (alcohol and other drug) consumption, AODR (alcohol and other drug related) interpersonal and societal problems, environmental-level prevention, social policy prevention approach, community-based prevention, school-based prevention, impact of policy or law, AOD laws, law enforcement, prevention through availability and accessibility, minimum drinking age laws, drinking and driving, ABC laws, alcoholic beverage distribution laws, location and density of AOD outlets, advertising, alcoholic beverage price, server intervention

## Abstract

Policy measures intended to control alcohol use and related problems have seldom been specifically targeted toward the entire group of young people between the ages of 18 and 25. Research evaluating these policies also tends not to focus on the 18–25 age group but, rather, on 18- to 20-year-olds or the adult population as a whole. Furthermore, some studies of alcohol control policies are cross-sectional and thus can offer only tentative information about the causes of results observed. Despite these limitations, the current literature does offer evidence for the effectiveness of particular alcohol control measures deserving of trials and further study among young adults. These measures affect the availability of alcohol, social messages about alcohol, and enforcement of current laws.

Alcohol use among young people is commonly associated with a number of ills, such as poor academic and work performance, community disruption, violence, injuries, and deaths. One way to prevent alcohol-related problems—among young people or the population as a whole—is to establish public and institutional policies that reduce overall alcohol consumption rates or reduce rates of high-risk drinking. This is commonly referred to as the “environmental approach” to alcohol problems—changing the community and policy environment to reduce drinking and its health and social problems (Wagenaar and Perry 1994). Alcohol control policies influence the availability of alcohol, the social messages about drinking conveyed by advertising and other marketing approaches, and the enforcement of existing alcohol laws (Babor et al. 2003). Availability of alcohol can be determined by policies that specify who can consume alcohol, where it can be consumed, and how and where alcohol is distributed and sold, as well as policies that affect the price of alcohol. Social messages are influenced by policies that regulate how alcohol is advertised and marketed (Toomey and Wagenaar 1999). Laws prohibiting underage drinking, the sale of alcohol to minors, or underage drinking and driving are a third component of the broader social environment that influences drinking by young adults.

Of the numerous alcohol control measures in effect at the National, State, local, and institutional levels, few specifically target the entire young adult age group—that is, those between the ages of 18 and 25. Some policies are aimed at young people under the legal drinking age of 21 but are less likely to affect young adults ages 21 to 25. Institutional policies on college campuses are intended to reduce alcohol consumption and alcohol-related problems among college students, the majority of whom are under the legal drinking age but, of course, have no effect on the many young adults who do not attend college. Other alcohol control efforts target the drinking behavior of the population as a whole, rather than that of specific subpopulations. Some of these policies may have a disproportionate effect on young adult drinkers compared with the rest of the population—for example, measures that address activity in bars and clubs, because young adults are more likely to patronize these establishments.

Not only have alcohol policy measures infrequently targeted the specific age group ages 18–25, researchers have seldom evaluated the effects of alcohol control policies specifically on this age group as a whole, although numerous studies have examined alcohol policy effects on the entire adult population, those under age 21, or college students. This article summarizes research on the effectiveness of alcohol control policies aimed at underage youth, college students, and the general population and, in particular, their effects on the drinking behavior of young adults.

## Alcohol Control Policies: Underage Youth

The primary and most-studied alcohol control measure relevant to underage youth is the establishment of the minimum legal drinking age (MLDA) at 21. Many well-controlled longitudinal studies have shown that an MLDA of 21 is effective in reducing alcohol consumption and traffic crashes among 18- to 20-year-olds, and some investigators have found that the age-21 MLDA is associated with reductions in other problems among underage youth, such as alcohol-related suicide and vandalism (Wagenaar and Toomey 2002). One study examined whether the age-21 MLDA had a spillover effect on the drinking behavior of 21- to 25-year-olds (O’Malley and Wagenaar 1991). This research found that college students who had been high school seniors in States with an MLDA of 18 drank more while in college than their counterparts who had been high school seniors in States with an MLDA of 21. High school graduates of the same age who were not attending college also drank more on average if they had been seniors in States with an MLDA of 18.

The age-21 MLDA has been effective in reducing teen drinking and alcohol problems with limited enforcement, which may illustrate the value of broad environmental approaches. Passage of the age-21 law established a norm that drinking by teenagers is inappropriate. This moral force of the law has some effect, even without active enforcement. In other words, many obey laws because it is the right thing to do, not just because of fear of enforcement penalties. Nevertheless, the law without enforcement is limited in its effects. Teenagers can easily obtain alcohol from both commercial outlets (e.g., bars and liquor stores) and social sources (e.g., friends, coworkers) (Wagenaar et al. 1996). Investigators have identified several policies and environmental strategies that strengthen MLDA laws and reduce underage access to alcohol (Komro and Toomey 2002)—for example, checking the compliance of commercial outlets by having a minor attempt to purchase alcohol under the supervision of a law enforcement officer. If an establishment sells alcohol to the underage person, penalties can be applied to both the server and the license holder. Studies have shown that compliance checks are effective in reducing sales to underage youth (Wagenaar et al. 2005) and indicate that compliance checks may affect drinking behavior (Holder et al. 2000).

Other policies targeting youth access to alcohol, such as beer keg registration and penalties for producing or selling false age identification documents, have not been evaluated in terms of their effects on access or drinking rates.

## Alcohol Control Policies: College Students

In recent years, an increasing number of colleges have implemented policies to reduce alcohol consumption and alcohol-related problems among their underage as well as legal-age students (Toomey and Wagenaar 2002). Examples of these institutional policies include establishing alcohol-free college residences and campuses, prohibiting self-service of alcohol at campus events, prohibiting beer kegs on campus, and banning sales or marketing of alcohol on campus.

Several studies have examined the effect of such college policies on the drinking behavior of college students. Large studies of nationally representative samples of college students have shown that students living in substance-free residences are less likely to engage in heavy episodic drinking (five or more drinks in one sitting for men, four or more for women), and underage students at colleges that ban alcohol are less likely to engage in heavy episodic drinking and more likely to abstain from alcohol (Wechsler et al. 2001*a*,*b*). College alcohol policies are less likely to have effects on students who live off campus than on, however, and are not likely to have any effect on the many young adults who do not attend college.

One limitation of the research on college alcohol policies is that students typically are surveyed at only one point in time—that is, these are cross-sectional studies, which offer little information as to cause and effect. Thus, this research cannot adequately address whether college prohibitions of alcohol use affect student drinking rates, or whether students who drink less choose residences and campuses that prohibit alcohol use. Longitudinal research is needed that specifically evaluates how *changes* in college policies affect drinking and alcohol problem outcomes on college campuses. One such longitudinal study which has been reported recently (Weitzman et al. 2004) examined the effects of an intervention designed to change alcohol policies affecting the college environment. This study showed no significant effects when all treatment sites were compared with comparison sites, but found some evidence that the intervention affected drinking and related risks at the five high-implementation colleges.

## Alcohol Control Policies: General Population

Alcohol control policies intended to affect the drinking behavior of the general population—including but not specifically targeting young adults—include, among other strategies, raising taxes on alcoholic beverages, limiting the number of alcohol establishments per geographic area, training the staff of alcohol outlets, and restricting alcohol marketing and advertising.

### Raising Prices

Of these policies, the effects of raising alcohol prices have been studied most intensely. The most common method of raising prices is to increase Federal, State, or local taxes on alcoholic beverages. Many investigators, using various designs and analytic methods, including high-quality longitudinal designs, have shown that as alcohol prices increase, alcohol consumption declines (Cook and Moore 2002). Studies show that underage youth are particularly sensitive to increased price, decreasing their alcohol consumption by a greater amount than older drinkers when prices increase (Chaloupka et al. 2002).

A few studies have examined the effects of alcohol prices specifically on college students and young adults (Chaloupka et al. 2002). One analysis showed that college students who faced higher alcohol prices were less likely to transition from abstainers to moderate drinkers and from moderate to heavy drinkers (Williams et al. 2005). Another study found that low sale prices were associated with higher heavy episodic drinking rates among college students (Kuo et al. 2003).

### Limiting the Number of Alcohol Outlets

States and cities often place a legal limit on the number of alcohol establishments in a neighborhood, town, or city as a strategy to reduce alcohol consumption and alcohol-related problems among the general population. Recent studies using advanced analytical methods show that a higher density of alcohol outlets is related to increased rates of homicides and assaults (Gorman et al. 2001; Lipton and Gruenewald 2002). Treno and colleagues (2003) evaluated how density of alcohol outlets affects driving after drinking among 15- to 20-year-olds, finding that higher alcohol outlet density is associated with greater prevalence of driving after drinking. Research also indicates that colleges with more neighborhood alcohol outlets experience more drinking and drinking-related problems (Weitzman et al. 2003; Wechsler et al. 2002). Because most studies of alcohol outlet density are largely cross-sectional, it is not certain that higher numbers of alcohol outlets actually cause increased alcohol consumption and related problems, and each individual study has limitations. Nevertheless, the increasing methodological sophistication of density studies, longitudinal studies of effects of sudden changes in density, and repeated findings of relationships between outlet density and alcohol use and alcohol problems provide growing evidence that outlet density may cause increased alcohol use and problems.

### Training the Staff

Some alcohol control policies that are aimed at the general population may have a greater effect on the drinking behavior of young adults than on drinking by older adults or underage youth. For example, policies that specifically target bars and nightclubs may affect young adults more than the rest of the population because young adults make up a greater proportion of bar and club clientele.

One frequently studied environmental strategy involves training servers, owners, and managers of bars and other alcohol establishments in responsible beverage service. For example, bar employees can be taught to recognize false age identification, to refuse sales to underage or obviously intoxicated patrons, and to offer food and nonalcoholic beverages to reduce patron intoxication levels. Studies have produced mixed results on the effectiveness of these training programs. Some programs result in changes in server attitudes and modest changes in server behaviors. But rates of illegal sales to underage or intoxicated patrons rarely change significantly following training (Toomey et al. 2001; Wagenaar et al. 2005). Some studies show that, at least within controlled environments such as the military, training may lead to lower intoxication levels among patrons (Saltz 1987). One study found a significant decrease in traffic crashes following implementation of a State law mandating server training (Holder and Wagenaar 1994). However, responsible beverage service training programs and laws vary greatly in quality (Mosher et al. 2002) and probably in effectiveness. No studies have assessed the effects of responsible beverage training on rates of alcohol use and related problems specifically among 18- to 25-year-olds.

### Restrictions on Marketing

Some localities have worked to restrict how alcohol is marketed to the public—for example, by limiting drink specials (such as happy hours or two-for-one promotions) at clubs, bars, and restaurants. Drink specials appear to be associated with increased alcohol consumption or drinking–driving, and some research indicates that drink specials on or near college campuses are associated with increased heavy episodic drinking among college students (Kuo et al. 2003).

## Comprehensive Approaches

Ultimately, sustained reductions in alcohol-related problems among young adults will require multiple environmental changes. Large-scale community trials have assessed the effects of comprehensive environmental approaches to reducing alcohol consumption and alcohol-related problems.

One community trial, Communities Mobilizing for Change on Alcohol (CMCA), specifically targeted a segment of the young adult population, 18- to 20-year-olds. The goal of CMCA was to mobilize communities to reduce youth access to alcohol through public and institutional policy changes. Fifteen communities were randomly assigned to the intervention or control condition, and a wide range of outcomes were measured before and after a 2½-year intervention period. In each of the seven intervention cities, community organizers conducted grassroots campaigns to influence local policy changes. Following community organizing efforts, fewer establishments in the intervention communities were likely to sell alcohol to underage youth, and fewer 18- to 20-year-olds in intervention sites reported providing alcohol to other underage youth compared with those in control communities. In intervention communities as compared with control communities, the proportion of 18- to 20-year-olds who tried to buy alcohol decreased 25 percent, the proportion who reported drinking in the last 30 days decreased 7 percent, and arrests for driving under the influence decreased 23 percent (Wagenaar et al. 2000*a*,*b*).

The Community Trials Project in California and South Carolina induced three communities to implement media advocacy, server training, restrictions on alcohol outlet density, and enforcement of underage sales and drinking–driving laws to reduce high-risk drinking and alcohol-related problems (Holder et al. 2000). The project’s interventions resulted in a significant decline in alcohol consumption, drinking–driving, traffic crashes, and assaults. The number of alcohol sales to underage youth also significantly decreased in the intervention communities compared with the comparison communities (Grube 1997). The Community Trials Project did not assess the effects of this array of environmental approaches on the rates of drinking and alcohol-related problems specifically among young adults.

Finally, the Complying with the Minimum Drinking Age project, using a 20-community time-series trial, tested the effects of enforcing laws governing sales to minors and of training alcohol outlet owners and managers. The propensity of alcohol retailers to sell to those under age 21 was measured biweekly over 4 years. Training had little effect, but enforcement significantly reduced sales to those under the legal drinking age (Wagenaar et al. 2005).

In summary, several community intervention trials demonstrated that various dimensions of alcohol policy affect important outcomes such as sales of alcohol, drinking, and indices of alcohol-related problems among teens and young adults.

## Future Directions

A substantial amount of research demonstrates the effectiveness of alcohol control policies aimed at teenagers, especially the legal drinking age of 21 and measures that take advantage of teens’ sensitivity to alcohol prices (e.g., raising taxes on alcohol). (For a discussion of the effectiveness of laws lowering the maximum legal blood alcohol content of drivers, see the sidebar.) The literature on alcohol control policies targeting the general adult population is extensive as well, providing support for raising alcohol taxes and prices and limiting the density of retail alcohol outlets. Recently, the alcohol policy environment as it specifically affects college students has been the subject of an increasing number of studies, indicating that schools with tighter controls on alcohol sales, marketing, and use experience lower rates of drinking and alcohol-related problems. The college studies to date have yielded mixed findings, however, probably because of the low number of controlled randomized or time-series trials and because some studies have used nonprobability samples at small numbers of colleges, which are not representative of college students as a whole.

**Reducing Drinking–Driving Among Young Adults: Effects of BAC Laws**

Traffic crashes are the leading cause of death among teens, and 32 percent of all drivers ages 16–20 who died in traffic crashes in 2003 had measurable alcohol in their blood; also, 51 percent of drivers ages 21–24 who died tested positive for alcohol (National Highway Traffic Safety Administration [NHTSA] 2004). One effective strategy to reduce drinking–driving is to lower the legal limit for allowable blood alcohol content (BAC) for drivers. In the past two decades, all States in the United States have adopted a BAC limit of 0.08 percent for adult drivers and have a BAC limit of zero, or slightly higher, for youth under age 21. These often are referred to as “zero tolerance” laws. (Most laws use a 0.02-percent limit rather than an absolute zero limit to allow for small measurement errors in BAC test instruments and to avoid challenges from youth who claim they have taken medication with small amounts of alcohol.)

Studies have found that laws setting the legal allowable BAC at 0.08 percent have resulted in 5-percent to 8-percent reductions in alcohol-related fatal traffic crashes among all drivers (Bernat et al. 2004; Dee 2001; Hingson et al. 2000; Voas et al. 2000). Laws setting the limit at 0.02 percent have led to a 19-percent reduction in drinking–driving and a 20-percent reduction in fatal traffic crashes among young drivers (Wagenaar et al. 2001; Hingson et al. 1994).

—Alexander C. Wagenaar

ReferencesBernatDHDunsmuirWTMWagenaarACEffects of lowering the legal BAC to 0.08 on single-vehicle-nighttime fatal traffic crashes in 19 jurisdictionsAccident Analysis & Prevention361089109720041535088610.1016/j.aap.2004.04.001DeeTSDoes setting limits save lives? The case of 0.08 BAC lawsJournal of Policy Analysis and Management201111282001HingsonRHeerenTWinterMLower legal alcohol limits for young driversPublic Health Reports10973874419947800781PMC1403574HingsonRHeerenTWinterMEffects of recent 0.08% legal blood alcohol limits on fatal crash involvementInjury Prevention610911420001087566610.1136/ip.6.2.109PMC1730627National Highway Traffic Safety Administration (NHTSA)Traffic Safety Facts 2003 Annual Report: Early EditionWashington, DCU.S. Dept. of Transportation2004VoasRBTippettASFellJThe relationship of alcohol safety laws to drinking drivers in fatal crashesAccident Analysis & Prevention3248349220001086875110.1016/s0001-4575(99)00063-9WagenaarACO’MalleyPLaFondCLowered legal blood alcohol limits for young drivers: Effects on drinking, driving, and driving after drinking behaviors in 30 statesAmerican Journal of Public Health9180180420011134489210.2105/ajph.91.5.801PMC1446683

Although very few studies have concentrated on the effects of the alcohol policy environment specifically on the entire population of 18- to 25-year-olds, both in college and in the work force, investigations of those in the 18–20 age group and of the general population 18 and over provide clear evidence that young adult drinkers are influenced by the alcohol policy environment. Young adult drinkers may be particularly susceptible to the influence of the policy environment, given that they drink more, on average, than other population groups (Substance Abuse and Mental Health Services Administration [SAMHSA] 2004) and are more likely to frequent social events and locations where drinking is common and heavy drinking often is overtly encouraged.

Controlled trials of specific alterations in the alcohol policy environment on college campuses and urban environments with high concentrations of young adults clearly are warranted, as are additional time-series studies of natural experiments with alcohol policies. However, even without the specific knowledge yet to be obtained from more controlled studies, enough is known to justify taking particular steps now to ameliorate the effects of alcohol problems among young people. The current state of knowledge supports a number of recommendations:

- Eliminate happy hours, free drinks, and other price promotions.
- Raise taxes on alcohol to increase the retail price.
- Limit the number and density of alcohol retail establishments.
- Enforce current statutes prohibiting sales of alcohol to those under age 21 and to anyone showing obvious signs of intoxication.
- Change social norms by reducing or eliminating the active promotion of drinking in settings were teens and young adults congregate, such as college campuses, music concerts, and sporting events.
